# Food frequency questionnaires developed and validated in Iran: a systematic review

**DOI:** 10.4178/epih.e2020015

**Published:** 2020-03-30

**Authors:** Arezoo Rezazadeh, Nasrrin Omidvar, Katherine L. Tucker

**Affiliations:** 1 Department of Community Nutrition, National Nutrition and Food Technology Research Institute, Faculty of Nutrition Sciences and Food Technology, Shahid Beheshti University of Medical Sciences, Tehran, Iran; 2 Department of Biomedical and Nutritional Sciences, University of Massachusetts Lowell, Lowell, MA, USA

**Keywords:** Food frequency questionnaire, Validity, Reproducibility, Systematic review, Iran

## Abstract

**OBJECTIVES:**

To systematically review and identify food frequency questionnaires (FFQs) developed for the Iranian population and their validation and reproducibility in order to determine possible research gaps and needs.

**METHODS:**

Studies were selected by searching for relevant keywords in the PubMed, Scopus, Science Direct, Google Scholar, SID, and Iranmedex databases, unpublished data, and theses in November 2016 (updated in September 2019). All English-language and Persian-language papers were included. Duplicates, articles with unrelated content, and articles only containing a protocol were excluded. The FFQs were categorized based on: (1) number of food items in to short (≤80 items) and long (>80 items) and; (2) the aim of the FFQ to explore total consumption pattern/nutrients (general) or to detect specific nutrient(s)/food group(s) (specialized).

**RESULTS:**

Sixteen reasonably validated questionnaires were identified. However, only 13 presented a reproducibility assessment. Ten FFQs were categorized as general (7 long, 3 short) and 6 as specialized (3 long, 3 short). The correlation coefficients for nutrient intake between dietary records or recalls and FFQs were 0.07-0.82 for long (general: 0.07-0.82 and specialized: 0.26-0.67) and 0.20-0.67 for short (general: 0.24-0.54 and specialized: 0.20-0.42) FFQs. Long FFQs showed higher validity and reproducibility than short FFQs. Reproducibility of FFQs was acceptable (0.32-0.89). The strongest correlations were reported by studies with shorter intervals between FFQs.

**CONCLUSIONS:**

FFQs designed for the Iranian population appear to be appropriate tools for dietary assessment. Despite their acceptable reproducibility, their validity for assessing specific nutrients and their applicability for populations other than those they were developed for may be questionable.

## INTRODUCTION

Diet is a major lifestyle-related risk factor for chronic disease. Therefore, methods of assessing dietary intake in epidemiological studies need to be evaluated. Food frequency questionnaires (FFQs) are the most commonly used tools to estimate usual dietary intake, especially in large epidemiological studies of nutrition [1,2]. FFQs are considered a “screener” method, developed to evaluate and rank the intake of nutrients or food groups to investigate associations between diet and disease [3].

FFQs are popular because they are less expensive, easier to use, and less time-consuming than other methods, and have the ability to capture long-term dietary intake [4]. However, despite their considerable advantages, FFQs may not be well-structured and appropriately used [5]. Factors such as respondents’ characteristics, the quantification of food intake (by portion size), and quality control and management of data may influence the accuracy of dietary intake assessment [6]. To categorize individuals accurately according to their nutrient intake, the validity and reproducibility of any FFQ should be assessed [7].

FFQs consist of a list of selected food items for which the respondent is asked to indicate how often they eat each item per day, week, or month. The list of foods may be chosen for the specific purposes of a study, and therefore may not assess respondents’ total diet. Furthermore, the food list may vary based on the ethnic, social, and cultural background of the population, and should be tailored to reflect those characteristics [2]. Therefore, the usefulness of FFQs depends on the appropriateness of the food list, which should reflect the usual food items or dishes consumed by the studied population. Furthermore, the specific accuracy of FFQs can be less than that of quantitative dietary assessment methods [8]. Accurate data on the amount of food and beverage consumption depends on assumptions for standardized portion sizes compatible with the amounts commonly consumed per serving in a culture or age/gender group [2,8]. Therefore, FFQs need to be adapted and validated for use in different specific contexts.

In Iran, the use of FFQs is relatively recent, dating from the early 2000s [9]. No comprehensive review has been conducted on FFQs developed and validated in Iran. Therefore, the present study aimed to systematically review FFQs developed and validated in Iran in order to identify possible research gaps and needs in this regard.

## MATERIALS AND METHODS

A systematic review was carried out by searching with relevant keywords or phrases, including “food frequency questionnaire,” “FFQ,” “reliability,” “validity,” and “Iran,” in the Medline, EMBASE, CINAHL, ProQuest, PubMed, Google Scholar, Scopus, Science Direct, SID, and Magiran (in Persian) databases, unpublished data, and thesis websites such as Irandoc (in Persian). Unpublished articles were obtained by contacting the authors to compare the food or nutrient items, as well as the questionnaire structure. The inclusion criteria were original human studies on FFQ validation performed on Iranian subjects published in the English or Persian languages. The reference lists of selected articles were checked to find additional related articles. If the full text was not provided for an abstract, the authors were contacted.

**Figure 1.**
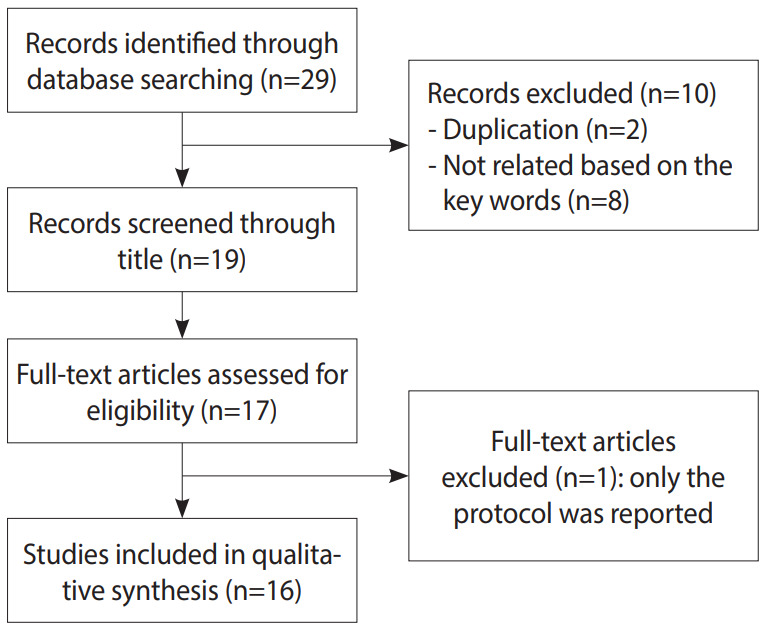
Flow diagram of the systematic review and meta-analysis process.

Initially, 29 articles were found. Two reviewers who were experts in the subject matter then analyzed the titles and abstracts of the selected articles to confirm their inclusion. Duplication was also checked and 19 articles remained. In the next step, one study [10] was excluded as it did not contain a full report and 2 unrelated articles (based on content) were also excluded (Figure 1). Finally, 16 papers met the criteria for the systematic review, based on the PRISMA (Preferred Reporting Items for Systematic Reviews and Meta-Analyses) checklist. The methodology for this systematic review was registered in the International Prospective Register for Systematic Reviews (in process; receipt code: 154722).

For each study, the following data were extracted: FFQ type, method of development, number of food items in the food list, questions on portion sizes, number of response categories for intake frequency, dietary reference method, and time interval between applications of the FFQ (for reproducibility), whether deattenuation and/or adjustment for energy was done, and number of nutrients evaluated. Data were categorized based on nutrients and food groups.

The questionnaires were categorized into general FFQs and specialized FFQs that were developed for assessing special nutrients or food groups. Furthermore, based on the number of food items in the food list, FFQs were classified as short (≤ 80 items) or long (> 80 items). This cut-off was based on a previous study [11] and arbitrary agreement between the authors. Validity (based on extracting the correlation coefficients between diet records (DRds)/recalls and FFQ estimates or agreement/disagreement between categories of mean/median/frequency of nutrient/food group consumption [cross classifications] between the FFQ and the reference method) and reproducibility (correlations between repeated administrations of the FFQ) were assessed. The values used to categorize the strength of correlations were based on a similar study by Wakai [12], which used the following criteria to categorize the strength of correlations: correlation coefficients 0.60 or more were considered high correlations, while correlation coefficients of 0.40-0.59, 0.30-0.39, < 0.30 were considered to indicate moderate, fair, and poor agreement, respectively. These cut-offs were used to define the validity of FFQs.

### Ethics statement

As the present study was a systematic review, no ethics statement was needed.

## RESULTS

Based on the literature search, 16 FFQs met the inclusion criteria (Table 1). They were all published, although in some cases missing information in the articles was identified through contacting the authors. No FFQ was found by searching unpublished data.

### Characteristics of food frequency questionnaires developed and validated in Iran

The detailed characteristics of FFQs, including the food list, presence of questions on portion/serving sizes, method of development, and type of questionnaire, are presented in Table 1. Most FFQs (n= 14) [13-26] were semi-quantitative, 2 were qualitative [9,27] and only one was considered quantitative [20].

Ten of the FFQs were general questionnaires that included food items from all food groups [11,13-21], of which 7 had a long format (89-189 food items in the list) and 3 were short (48-80 food items). They were all validated for adults, except for 2 that were specially designed and validated for elderly individuals [17,18].

The other 6 FFQs were categorized as specialized FFQs, which had been developed to estimate the intake of specific nutrients (vitamin A, calcium, folate, pyridoxine and cobalamin, and sodium) or food groups (fruit and vegetables). Among the specialized FFQs, 4 had a long format [23,25,26,28], while the other 3 were short [9,24,27]. All the specialized FFQs were developed for adults, except for an FFQ on calcium, which was developed for school-age children [24], and an FFQ on sodium sources, which was developed for those aged 6 years and above [26].

The approach used to develop the food list in 7 FFQs was databased [9,11,14,15,22,23,26], in which food items for the FFQ food list were chosen according to data from DRds or recalls. Only one FFQ [27] used an experience-based method by selecting food items based on the opinions of an expert panel regarding the most common food items or foods usually consumed in the study population. Three were developed through a combination of databased and experience-based approaches [13,18,24]. In addition, 2 questionnaires were modified versions of the Tehran Lipid and Glucose Study (TLGS) FFQ, in which new food items were added using an experience-based approach [17,19], and 3 questionnaires were translated and modified versions of original questionnaires from other countries [20,25,28].

### Comparison of food items in Iranian food frequency questionnaires

Long-format questionnaires, except for the dish-based FFQs [16,20], included food items based on food groups. Common food items in Iranian FFQs included 9 items for breads and cereals (4 main traditional types of bread, rice, spaghetti, noodles, biscuits, and crackers); 4 dairy products without considering the fat content and processing type (milk, yogurt, cheese and *doogh*/yogurt drink); 17 fruits (bananas, oranges, apples, pears, plums, peaches, apricots, nectarines, cherries, melons, watermelon, grapes, pomegranates, canned fruit, dates, dried fruits, and fruit juice); 16 vegetables (dark green vegetables, lettuce, tomato, cucumber, eggplant, squash, onion, garlic, carrot, bell pepper, green pepper, corn, potato, cabbage, green beans, and green peas), meat, poultry, fish, and eggs (each as a single item); 6 legumes (lentils, chickpeas, split peas, dried beans, soybeans, and broad beans); 5 nuts (pistachios, almonds, peanuts, hazelnuts, and walnuts), 2 seeds (sunflower and pumpkin); 4 items for oils and fats (hydrogenated vegetable oil, vegetable oil, olive oil, and butter); and 1 sweet (chocolate). Pizza, although it is considered a dish, was included in 2 food items–based FFQs [14,15,17]. In addition, although the common items listed above were based on dietary pattern(s) of the population under study, other items were added to the FFQs’ food lists, such as traditional dairy foods, such as camel *doogh* and *aqaran*, smoked and salted fish, as well as yellow oil in the FFQ developed for the Golestan cohort study [13].

Of the developed and validated questionnaires, one was dishbased [16], and another included a combination of common general food items and common dishes consumed by Iranians [20].

### Validity and reproducibility of food frequency questionnaires in Iran

In most studies, the validity of the developed FFQ was evaluated by assessing the correlation of dietary intake estimates against 24-hour dietary recalls (24hDRs) [9,11,12,18,19,22,23], DRds [14,15,17], or nutrient biomarkers [9,13,20,22,24,27] as reference methods (Table 2). The correlation coefficients between measured nutrients from FFQs and reference measures were between 0.07 to 0.82 in the general FFQs and 0.20 to 0.67 in the specialized FFQs. All proposed questionnaires appeared valid, except for one [14]. Six studies reported high correlations (r≥ 0.60) between FFQ estimates and reference measure(s) [13-15,20,23,25], 11 presented moderate correlations (r = 0.40-0.59) [11,13-15,18-21,23-25], 9 had fair correlations (r=0.30-0.39) [14,15,18-21,23-25], and 6 had poor correlations (r< 0.3) [9,16,19-21,23] for some food groups/nutrients derived from FFQs.

The range of correlation coefficients for short FFQs (r= 0.20-0.54; general: 0.24-0.54; specialized: 0.20-0.42) was slightly lower and narrower than that of long FFQs (r= 0.07-0.82; general: 0.07-0.82; specialized: 0.26-0.67).

Some studies demonstrated benefits from assessing additional validity method(s) such as cross-classification to discover the ability of the developed FFQ to accurately classify subjects by groups of intake (data not shown) [11,14,15,19-21,23,24]. Statistically, complete agreement was reached when subjects were in the same intake category according to 2 methods; adjacent agreement was when participants were categorized into roughly similar groups (e.g., the second quartile of intake by FFQ but the third quartile of intake by the reference method); and complete disagreement was observed if subjects were classified into the lowest intake class by FFQ and highest intake class by the reference method or vice versa. Complete agreement observed between food/nutrient items from 20% to 91% while disagreements was reported sparingly, ranging from 0% to 19%. A subanalysis by gender revealed that the mean agreement was higher in men (31.0-68.3%) than in women (27.0-54.1%), and the frequency of complete disagreement was similar in both (men: 0-21%; women: 0-25%). In most items, complete and adjacent agreement was observed and disagreement was seen mostly in food that were consumed seldomly (e.g., pickles) and some micronutrients (vitamins D, B_12_, B_3_, beta-carotene, calcium, and iron).

One study only tested agreement by Bland–Altman scatter plots [19], and found that almost all individuals showed consistent variations across levels of intake, and only a few participants fell outside the limits of agreement for energy and macronutrients.

Reproducibility of all but 4 questionnaires had been assessed [19,20,29,30]. To evaluate reproducibility, the FFQs were completed twice within an interval of 2 weeks [21,22] to 1 year [14,15]. In one study [13], 4 FFQs were completed at a 2-month interval. All studies had acceptable reproducibility, with significant correlations between nutrient intake values (ranging from 0.32 to 0.89). The strongest correlations were reported by studies with shorter intervals between the 2 FFQs [13,21,22].

## DISCUSSION

Sixteen FFQs (10 long and 6 short) that were developed and validated for the Iranian population were identified. Most of the questionnaires were reasonably valid and reproducible (16 valid and 13 reproducible). However, relatively poor validity was observed in FFQ estimates for several food groups and nutrients. FFQs with a long format had slightly better estimations of nutrient intake. Almost all FFQs used for dietary assessment in the Iranian population were developed and validated for adults, except for one developed to assess calcium intake in children [24] and 2 developed to assess dietary intake in the elderly [18,19].

The Iranian FFQs were mostly validated against a dietary reference method (24hDRs [9,11,13,19-21,23,24,26] or DRds [11,14,15,16,18,21,23]) and, in some cases, biomarkers [9,14,21,23,25,28]. The range of correlations for nutrient intake estimates between the dietary reference method and Iranian FFQs were similar to those reported for Japanese FFQs (0.42 to 0.52) [12], but lower than that reported for Western countries (0.60 to 0.74) [29]. A systematic review of FFQs developed and validated for the Brazilian population showed that the correlations reported between FFQs and the reference method were equal to or less than 0.4 for certain nutrients and above 0.4 for others [30]. As Wakai [12] stated, “This variation may be due to differences in the number of food items, ability to recall intake frequencies and portion sizes, the wording of questions in the FFQ, and between-person variations in consumption among food groups.” For example, in short FFQs, smaller numbers of food items in the food list may lead to weaker correlations between dietary recalls and FFQs.

None of the reviewed studies reported the response rates of FFQs. Thompson & Subar [2] noted that although longer FFQs can better reflect the total diet, short FFQs can have higher response rates and are less burdensome for respondents. Additionally, the inclusion of mixed dishes and traditional foods in some Iranian FFQs may have led to higher variation, even in long FFQs, while list-based questionnaires could not reflect information on the items used by various subcultures, including detailed information on food preparation, cooking methods, and additives [3].

Among all the Iranian validation studies that were reviewed, only three explored correlations between estimations of food group intake based on a reference method (DRd or 24hDR) and those made based on FFQs [23]. Mohammadifard et al. [21] also investigated the effects of season on these correlations and found considerable correlations between dietary recalls and FFQs for total fruit/vegetable intake in both hot and cold seasons (0.60 to 0.62). According to Wakai [12], variation in food group correlations between the reference method and the FFQ depends on the definition of portion size and the number of food items listed in the FFQ. Food items with an easier and more understandable portion size (for example 1 medium raw carrot in comparison with 1/2 cup of cooked vegetables) result in higher validity.

Questionnaires designed to assess the intake of a single nutrient had different degrees of validity, depending on the type of nutrient studied. One FFQ that was specifically developed to assess folate, pyridoxine, and cobalamin intake had acceptable validity, with correlations between DRds and FFQ ranging from 0.51 to 0.67 [25]. Additionally, the validity of a questionnaire designed specifically for calcium intake in children was close to the acceptable range (r= 0.42) [24]. Similar to the Iranian FFQ for calcium [24], a systematic review of FFQs developed for calcium intake in children found good validity in children 12 months to 36 months of age, and concluded that semi-quantitative FFQs were valid and reproducible for assessing dietary intake at the group level [32]. In addition, the findings of a systematic review on dietary assessment methods in children 11 years of age or younger suggested that FFQs can be more reliable and valid than other dietary assessment methods (i.e., 24hDRs and DRds) in this age group [32]. However, another review of FFQs developed for children and adolescents concluded that FFQs were the most valuable method for assessing total energy intake in children aged 4 years to 11 years, compared to total energy expenditure measured by double-labeled water. In contrast, for younger children aged 6 months to 4 years, weighed DRds provided the best estimate, whereas diet history provided better estimates for adolescents aged ≥ 16 years [33].

Furthermore, for some nutrients, respondents’ gender was associated with the strength of the correlations; for example, the correlations for beta-carotene, vitamins A, and C were lower for women than men in the TLGS [14,15]. This finding is remarkable given the presumption that women usually report intake more accurately than men, due the fact that they are usually responsible for food preparation and can provide more accurate response(s) [34]. In contrast, one study reported considerably weaker correlations for energy, fat, and micronutrients in men than in women (0.07-0.19 vs. 0.76-0.81) [16].

This review reported all available FFQs validated in the Iranian population, which may also be useful for populations in other Middle Eastern countries; however, it faced with some limitations that should be considered. The results of various studies were not completely comparable due to differences in the micronutrients or macronutrients studied for validation. Additionally, the FFQs differed in terms of the number of food items included and the purpose of their development (measuring specific nutrients, populations, or diseases), which may influence their interpretation.

## CONCLUSION

The FFQs designed for the Iranian population appear to be appropriate tools for dietary assessment; however, their validity for assessing specific nutrients and their applicability for populations other than those they were developed for may be questionable. Nonetheless, there was a large amount of commonality among the general FFQs. Therefore, choosing the proper FFQ for a specific population within Iran should be done with caution, and an analysis of their characteristics may be necessary. The lack of a general FFQ that can be used at the national level in Iran while being adjusted according to the dietary patterns of different ethnic and cultural groups is a gap that needs to be filled by future studies. Furthermore, the fact that few FFQs were developed for elderly individuals and children makes it difficult to judge their generalizability and usefulness for those age groups.

## Figures and Tables

**Table 1.** Characteristics of FFQs developed and validated in Iran

Study			Objective of the validation study	No. of food items	FFQ type	No. of response categories for intake frequency	Questions on portion size	Method of development of food list^1^	Quality control
General FFQ									
	Long form								
		Malekshah et al., 2006 [13]	To be used in the prospective cohort study (Golestan Cohort Study) in a population at high risk for esophageal cancer in northern Iran	158	Semi-quantitative	5	No (DPS is provided)	Experience & data-based	Interviewed at the subjects’ home and in their native language by trained interviewers and checked by a nutritionist for completeness
									Questionnaires were reviewed to identify incomplete and illogical response
									Participants were interviewed by different personnel at different days of recall to reduce interviewer bias
		Mirmiran et al., 2010 [14]	To capture the dietary practices of the study participants in TLGS	168	Semi-quantitative	5	Yes: 1 (DPS is provided)	Data-based	Trained nutritionists assisted in completing and rechecking and data entry of the assembled questionnaires
		Esfehani et al., 2010 [15]							
		Nematy et al., 2013 [16]	To assess nutrient intakes of Iran urban population	160	Semi-quantitative	8	Yes: 1 (DPS is provided)	Data-based	Not mentioned
		Ebrahimi-Mameghani et al., 2014 [17]	To approve content validity of TLGS FFQ for use in Tabriz city, northeast of Iran	189	Semi-quantitative	5	Yes: 1 (DPS is provided)	Modified version of TLGS questionnaire & added items were experienced-based	FFQs were completed by one trained nutritionist
		Malekahmadi et al., 2016 [18]	To estimate antioxidants intakes by the elderly in epidemiological studies	89	Semi-quantitative	5	Yes: 1 (DPS is provided)	Data & experience-based	A pilot study was conducted
									The questionnaires were rechecked for missing and unfilled items by trained nutritionists
		Bijani et al., 2018 [19]	For using in a cohort study (Amirkola Health and Aging Project of older people in the North of Iran)	138	Semi-quantitative	5	Yes: 1 (DPS is provided)	Modified version of TLGS questionnaire & experienced base for added items	Not mentioned
		Toorang et al., 2019 [20]	To develop a Farsi version of DHQ and validate it for epidemiological studies in Iranian population	146 (including Iranian mixed dishes)	Semi-quantitative (quantitative for some items)		Yes: 1-4 (DPS is provided)	Translated and modified version of the DHQ of the National Cancer Institute of America	Not mentioned
	Shot form								
		Mohammadifard et al., 2015 [21]	To validate a simple FFQ for assessing selected food items in epidemiological studies (specifically Isfahan Healthy Heart Program) with a large sample size as well as community trials	48	Semi-quantitative	6	Yes: 1 (DPS is provided)	Short form approach from its long format questionnaire used in Countrywide Integrated Non-communicable Disease Intervention program	Trained nutritionists assisted in completing and rechecking and data entry of the assembled questionnaires
									Excluding all the under- and overreportings of dietary intake (daily energy intake <800 or > 5,000 kcal)
									The subjects were followed by phone to verify and complete self-reported DRd
		Sharifi et al., 2016 [22]	To evaluate the reliability and validity of energy and macronutrient intake by a short FFQ in pregnant women	61	Semi-quantitative	10	Yes: 1 (DPS is provided)	Data-based	Pilot study
		Nikniaz et al., 2017 [11]	To administer a new short FFQ to assess the dietary practices (food patterns and nutrient intakes) of the study participants in epidemiological studies (e.g., Life style Promotion Project)	80	Semi-quantitative	4	Yes: 1 (DPS is provided)	Data-based	A pilot study for clarity and comprehensiveness of the FFQ was conducted
Specialized FFQs: single food groups or nutrients									
	Long form								
		Mohammadifard et al., 2011 [23]	To assess fruits and vegetable consumption in adults residing in the city of Isfahan	110 fruits and vegetables	Semi-quantitative	5	Yes: 1 (DPS is provided)	Data-based	Pilot study for clarity and comprehensiveness of FFQ
									The participants were followed by phone to verify and complete self-reported DRd
		Pirouzpanah et al., 2012 [25]	To evaluate the validity of folate, pyridoxine and cobalamin estimates in breast cancer patients	126 folate, pyridoxine & cobalamin	Semi-quantitative	25 questions about food preparation and 25 fields for open-ended questions	Yes (set of color photographs and customary household utensils were used to depict different portion sizes.)	Modified and shortened version of health habits and history questionnaire (35)	Not mentioned
		Pirouzpanah et al., 2014 [28]							
		Mohammadifard et al., 2016 [26]	To develop simple and accurate tools for salt consumption assessment	136 major sodium sources	Semi-quantitative	9	Yes: 1 (DPS is provided)	Data-based	Trained nutritionists assisted in completing and rechecking and data entry of the assembled questionnaires
	Shot form								
		Omidvar et al., 2002 [9]	To serve SH-FFQ as a simple screening tool for vitamin A status in women of childbearing age in an area where overt hypovitaminosis A is rare but mild deficiency is probably common	20 vitamin sources	Qualitative	5	No	Data-based	2-d course in dietary interviewing prior to the study and frequent supervision during the study
		Zeyninejad et al., 2015 [24]	To estimate calcium intake in 9-13 yr old students in the city of Tehran	56 calcium sources	Semi-quantitative	6	Yes: 1 (DPS is provided)	Data & experience-based	Two nutritionists were trained in a 7-d pilot study
									The same nutritionist interviewed the same subject in different stages of the study
									For 24hDR recorded information was reviewed to confirm the entries, and add possible forgotten items
		Hadi et al., 2017 [27]	To assess gluten intake in patients with ulcerative colitis	40 gluten sources	Qualitative	7	No	Experience-based	Not mentioned

FFQ, food frequency questionnaire; DHQ, diet history questionaire; DPS, default portion size; 24hDR, 24 hours recall; TLGS, Tehran Lipid and Glucose Study; SH-FFQ, short-food frequency questionnaire; DRd, diet records.

1 The first is the “experience-based” approach, in which experienced dietitians and/or epidemiologists select food items for the questionnaire; “Data-based” approach: Food items for FFQs are selected based on data from diet records so as to encompass defined percentages of the intakes of target nutrients; Short-version” approach: In this method, a long FFQ is shortened by omitting food items.

**Table 2.** Summary of validation studies for FFQs developed in Iran

Study			No. of food items	Participants	Correlation coefficients between dietary reference methods and FFQs (validity)					Correlation coefficients between FFQs (reproducibility)	
					Dietary reference method & its duration	No. of nutrients	Adjustment for energy	Energy adjustment and deattenuation	Nutrients (median [range])	Nutrients (median [range])	Interval between 2 FFQs
General FFQs											
	Long form										
		Malekshah et al., 2006 [13]	158	142 (57 urban, 85 suburban/rural) 49 men, 89 women	Twelve 24hDR (2 continues days)	3 macronutrients and FA profile 4 micronutrients (vitamins C, A, E, and beta-carotene)	No	For vitamin E (α-tocoferol) intake and serum level	0.49-0.82	0.66-0.89	4 FFQ 2 mo apart
		Mirmiran et al., 2010 [14]	168	132 (61 men, 71 women) 20-70 yr, 1 district in the city of Tehran	12 d DRd and 6 biomarkers (plasma cholesterol, retinol, beta-carotene, α-tocopherol, urine nitrogen and K)	3 macronutrients and FA profile, 12 micronutrient (vitamins C, A, D, beta-carotene, B1, B2, folate, Zn, Mg, Ca, P)	Yes	Yes	If significant: 0.33-0.71	0.39-0.79	2 FFQ 1 yr apart
		Esfahani et al., 2010 [15]									
		Nematy et al., 2013 [16]	160	156 subjects, aged 20-69 yr five major cities of Iran (Tehran, Tabriz, Mashhad, Isfahan, and Shiraz)	18 DRd (3 consecutive days each, including 1 weekend monthly)	1 macronutrient (protein) and 4 micronutrients (vitamins A, E, folate, K)	No	No	Only significant for carbohydrate =0.22	0.33-0.67	2 FFQ 4 mo apart
		Ebrahimi-Mameghani et al., 2014 [17]	189	30 healthy aged 20-60 yr living in Tabriz, East Azerbaijan	-	-	-	-	-	0.59-0.60	2 FFQs 2 mo apart
		Malekahmadi et al., 2016 [18]	89	185 elderly people (99 women and 86 men) aged 60-75 yr living in Isfahan	18 DRd (3 d every 2 mo)	Zn, selenium, carotenes, vitamins C and E	Yes	No	0.38-0.55	0.47-0.58	2 FFQs 3 mo apart
		Bijani et al., 2018 [19]	138	200 men and women aged ≥60 yr	Two 24hDR	3 macronutrients and FA profile, 19 micronutrients (vitamins A, E, C, B groups, iron, Zn, cupper, selenium, manganese Mg, Ca, P)	No	No	If significant: men: 0.25-0.53; women: 0.26-0.71	-	-
		Toorang et al., 2019 [20]	146	244 healthy adults (106 women and 138 men) aged 19-60 yr	One 24hDR in every season	3 macronutrients, 16 micronutrients (vitamins A, C, B groups, iron, Zn, cupper, selenium, sodium manganese Mg, Ca, K)	Yes	Yes	Men: 0.13-0.60	-	-
									Women: 0.07-0.58		
	Short form										
		Mohammadifard et al., 2015 [21]	48	264 healthy adults aged ≥ 41 yr old from 3 district central of Iran (Isfahan, Najafabad, and Arak)	Single 24hDR and 2 DRd (including 2 wk days and 1 weekend during a week)	-	No	No	If significant: 0.24-0.48	0.49-0.67	2 FFQs 2 wk apart
		Sharifi et al., 2016 [22]	61	553 pregnant women, aged 18-40 yr (in 3rd trimester)		-	No	No	Only content validity was evaluated	0.51-0.99	2 FFQs 2 wk apart (just filled by 20 participants)
		Nikniaz et al., 2017 [11]	80	180 subjects (93 men and 87 women), aged 15-65 yr	A single 24hDRs and 2 DRds for 3 non-consecutive days	3 macronutrients and FA profile, 19 micronutrients (vitamins A, E, C, D, B1, B3, iron, Zn, selenium)	Yes	Yes	Deattenuated correlation coefficients for all items: 0.54	Men: 0.43-0.77	2 FFQs 1 mo apart
										Women: 0.41-0.71	
Specialized FFQs: single food groups or nutrients											
	Long form										
		Mohammadifard et al., 2011 [23]	110 fruits and vegetables	123 healthy adults in Isfahan	Two 24hDR and 4 DRds (1 d recall, and 2 d of DRds in fall/winter (cold season) and spring/summer (warm season)) and three plasma biomarkers (vitamin C, retinol and betacarotene)	3 micronutrients (vitamin C, beta-carotene and retinol)	Yes	No	If significant: 0.26-0.62	If significant: 0.32-0.85	2 FFQs 6 mo apart
										If NS: 0.16-0.85	
		Pirouzpanah et al., 2012 [25]	136 folate, pyridoxine & cobalamin sources	149 women 30-69 yr of age, diagnosed with malignant breast tumor	No - biomarker reference method was used	3 micronutrients (folate, pyridoxine and cobalamin)	Yes	For energy and some nutrients	Folate: 0.61	-	1 FFQ as a reference method
		Pirouzpanah et al., 2014 [28]							Pyridoxine: 0.51		
									Cobalamin: 0.67		
		Mohammadifard et al., 2016 [26]	136 Na containing items (11 groups)	219 healthy subjects aged≥6 yr	The validity of FFQ: by twelve 24hDRs (monthly during a year) and 24 hr urine Na excretion	Na, K and Cr (24 hr urine and spot urine), fasting blood sample: FBG, serum lipids, Na, K and Cr	NR	NR	NR	NR	2 FFQs 1 yr apart
					Content validity: by expert panel including 10 nutritionists				CVI>0.79 for 11 food groups		
	Short form										
		Omidvar et al., 2002 [9]	20 vitamin A sources	187 healthy women 15-49 yr of age, from urban and rural areas of Marand district in East Azerbaijan.	2 d 24hDR and biomarker (serum retinol)	1 micronutrients (vitamin A)	No	No	Serum retinol: 0.20	-	-
									24hDR: NS		
		Zeyninejad et al., 2015 [24]	56 Ca sources	184 children aged 9-13 yr (90 girls and 94 boys)	Five 24hDR over a month period (four week days and one weekend)	1 micronutrients (Ca)	No	-	0.42	0.65	2 FFQs 1 mo apart
		Hadi et al., 2017 [27]	40 items (gluten containing food items)	-	10 expert panelists	Only content validity was assessed	No	No	CVR=0.69	Cronbach’s alpha: panelists=0.76 patients=0.78	3 FFQ 1 mo apart
					15 patients with ulcerative colitis				CVI=0.92		

FFQs, food frequency questionnaires; FA, fatty acid; Zn, zinc; Mg, magnesium; Ca, calcium; P, phosphorus; Na, sodium; K, potassium; Cr, creatinine; CVR, content validity ratio; CVI, content validity index; NS, non significant’; NR, not reported; 24hDR, 24 hour recall; DRd, dietary record; FBG, fasting blood glucose.
